# Role of acidosis-sensitive microRNAs in gene expression and functional parameters of tumors *in vitro* and *in vivo*

**DOI:** 10.1016/j.neo.2021.11.005

**Published:** 2021-11-13

**Authors:** Mandy Rauschner, Thea Hüsing, Luisa Lange, Kristin Jarosik, Sarah Reime, Anne Riemann, Oliver Thews

**Affiliations:** Julius Bernstein Institute of Physiology, University of Halle-Wittenberg, Magdeburger Str. 6, Halle (Saale) 06112, Germany

**Keywords:** Acidosis, microRNA, Gene expression, Migration, Proliferation, Brip1, BRCA1 interacting protein C-terminal helicase 1, Clspn, claspin, Crem, cAMP responsive element modulator, Dnajc25, DnaJ heat shock protein family (Hsp40) member C25, Ercc6l, ERCC excision repair 6 like, spindle assembly checkpoint helicase, Fstl1, follistatin like 1, Gls2, glutaminase 2, Ikbke, inhibitor of kappa light polypeptide gene enhancer in B-cells, kinase epsilon, Il6r, interleukin 6 receptor, MIBG, meta-iodobenzylguanidine, miRNA, microRNA, Per3, period circadian clock 3, Rif1, replication timing regulatory factor 1, Tlr5, toll-like receptor 5, Txnip, thioredoxin interacting protein

## Abstract

**Background:** The acidic extracellular environment of tumors has been shown to affect the malignant progression of tumor cells by modulating proliferation, cell death or metastatic potential. The aim of the study was to analyze whether acidosis-dependent miRNAs play a role in the signaling cascade from low pH through changes in gene expression to functional properties of tumors *in vitro* and *in vivo*.

**Methods:** In two experimental tumor lines the expression of 13 genes was tested under acidic conditions in combination with overexpression or downregulation of 4 pH-sensitive miRNAs (miR-7, 183, 203, 215). Additionally, the impact on proliferation, cell cycle distribution, apoptosis, necrosis, migration and cell adhesion were measured.

**Results:** Most of the genes showed a pH-dependent expression, but only a few of them were additionally regulated by miRNAs *in vitro* (Brip1, Clspn, Rif1) or *in vivo* (Fstl, Tlr5, Txnip). Especially miR-215 overexpression was able to counteract the acidosis effect in some genes. The impact on proliferation was cell line-dependent and most pronounced with overexpression of miR-183 and miR-203, whereas apoptosis and necrosis were pH-dependent but not influenced by miRNAs. The tumor growth was markedly regulated by miR-183 and miR-7. In addition, acidosis had a strong effect on cell adhesion, which could be modulated by miR-7, miR-203 and miR-215.

**Conclusions:** The results indicate that the acidosis effect on gene expression and functional properties of tumor cells could be mediated by pH-dependent miRNAs. Many effects were cell line dependent and therefore do not reflect universal intracellular signaling cascades. However, the role of miRNAs in the adaptation to an acidic environment may open new therapeutic strategies.

## Introduction

Tumor acidosis with extracellular pH (pH_e_) values down to 6.0, which is a common feature of human and experimental tumors [1], has been shown to modulate the malignant potential of tumors independently from concomitant hypoxia. The extracellular acidification, which shifts the pH_e_ from 7.2–7.4 in normal tissues to 6.1–7.0 in tumors, results from the anaerobic metabolism resulting from hypoxia but also from the feature of tumor cells switching to glycolytic metabolism even in the presence of oxygen ("Warburg" effect) [2]. Even though tumor cells have potent mechanisms to maintain the intracellular pH constant, the accumulation of protons in the interstitial space has been shown to modulate the cellular metabolism and mitochondrial function [3]. In parallel, acidosis modulates different functional properties of tumors affecting the malignant potential. The acidic micromilieu promotes the invasiveness and migration of tumors [4,5] and by this may affect the formation of metastases [6], [7], [8]. It is also known that the tumor pH affects tumor suppressor genes and oncogenes which in turn can modulate proliferation and apoptotic or necrotic cell death [9,10].

The mechanism by which the extracellular proton concentration may influence gene expression or functional properties of cells has been discussed controversially and is yet not fully understood. After sensing of the extracellular pH by, for instance, acid-sensitive ion channels and ion transporters or G-protein coupled H^+^-sensors [11,12] different intracellular signaling cascades, such as mitogen-activated protein kinases (e.g., ERK1/2, p38, JNK) [13], ROS generation [6] or Rho/Rho-kinase pathway [13,14], have been discussed to induce pH-dependent changes of gene expression. Small non-coding RNAs (miRNAs), which can regulate the expression of different target genes and thereby affect the functional behavior of tumor cells (proliferation, migration, cell adhesion, apoptotic or necrotic cell death) [15], might represent another possible mechanism. Previous studies revealed that extracellular acidosis modulates the expression of several miRNAs *in vitro* and *in vivo* [16]. Four miRNAs were consistently changed in different tumor lines: miR-7 (up-regulation), miR-183, miR-203 and miR-215 (down-regulation). In parallel, NGS and qPCR analyses indicated a large number of genes to be regulated cell line-independently under acidic conditions in cell culture (136 genes) and in experimental tumors (287 genes) [17]. Several of these acidosis-regulated genes have been predicted to be putative targets of the miRNAs mentioned above, for example Clspn (miR-7), Fstl1 (miR-203), Gls2 (miR-203), Il6r (miR-183, miR-203), Rif1 (miR-215) or Txnip (miR-183).

In accordance with this background, the aim of the present study was to test whether pH-dependent miRNAs are involved in the acidosis-induced changes of gene expression and furthermore in functional alterations of tumor cells, such as proliferation, cell cycle distribution, apoptosis and necrosis, migration and adhesion. Therefore, tumor cells were transfected with mimics or inhibitors (antagomirs) of the respective miRNA and exposed either to pH 7.4 or 6.6. Since miR-7 was up-regulated and miR-183, miR-203 as well as miR-215 were down-regulated the acidosis effect was simulated by overexpression of miR-7, and reduced expression of miR-183, 203 and 215 at pH 7.4. A second series of experiments was used to test whether the acidosis-induced effects could be counteracted by a lowering of miR-7 expression or overexpression of the other miRNAs (at pH 6.6). In the cells gene expression, proliferation, cell death, migration and adhesion was measured. For *in vivo* experiments, the transfected cells were subcutaneously implanted forming experimental tumors with a subsequent analysis of gene expression and functional parameters as tumor growth and cell proliferation.

## Materials and methods

### Cell lines

All studies were performed with two tumor cell lines of the rat: (a) subline AT1 of the Dunning prostate carcinoma R3327 (CLS # 500121, CLS GmbH, Eppelheim, Germany) and (b) Walker-256 mammary carcinoma (ATCC # CCL-38, LGC Standards GmbH, Wesel, Germany). Both cell lines were cultured at room air containing 5% CO_2_ in RPMI medium supplemented with 10% fetal calf serum (FCS) and for Walker-256 cells additionally with 10 mM L-glutamine, 20 mM HEPES, 0.15% NaHCO_3_. For the experiments, cells were incubated under serum starvation for 24 h in medium buffered with NaHCO_3_, 10 mM MES (morpholinoethanesulfonic acid) and 10 mM HEPES, with pH adjustment either to pH 7.4 or 6.6 with 1 N HCl.

### *In vivo* tumor models

Solid tumors of AT-1 cells were induced *in vivo* in male Copenhagen rats (body weight 115–292 g) and Walker-256 tumors in Wistar rats (body weight 165–231 g), housed in the animal care facility of the University of Halle. All experiments had previously been approved by the regional animal ethics committee and were conducted in accordance with the German Law for Animal Protection and the UKCCCR Guidelines [18]. Solid tumors were induced by heterotopic injection of cell suspension (6-8 × 10^6^ cells/0.4 ml isotonic saline) subcutaneously into the dorsum of the hind foot. Tumor volumes were determined by measuring the three orthogonal diameters with a caliper and with the formula: V=d_1_•d_2_•d_3_•π/6. Tumors were investigated when they reached a volume of 0.40–1.68 ml.

In order to induce a more pronounced tumor acidosis *in vivo*, metabolic acidosis was intensified by treating tumor-bearing animals with a combination of inspiratory hypoxia and meta-iodobenzylguanidine (MIBG) which forces glycolytic metabolism [19]. Therefore, animals received a MIBG injection (20 mg/kg b.w., i.p. dissolved in isotonic saline) and were then housed in a hypoxic atmosphere containing 10% O_2_ and 90% N_2_ for 24 h. This procedure reduced the extracellular pH in AT1 tumors from 7.02 ± 0.04 to 6.48 ± 0.08 and in Walker-256 tumors from 7.16 ± 0.03 to 6.65 ± 0.07 [16]. After 24 h animals were sacrificed, tumors were surgically removed, minced and total RNA was extracted using TRIzol reagent (Thermo Fisher Scientific, Waltham, MA, USA), while protein was isolated by using CST cell lysis buffer (20 mM Tris-HCl (pH 7.5), 150 mM NaCl, 1 mM EDTA, 1mM EGTA, 1% Triton, 2,5 mM sodium pyrophosphate, 1mM ß-glycerophosphate, 1 mM Na_3_VO_4_, protease inhibitor cocktail).

### miRNA transfection

In order to assess the impact of pH-dependent miRNAs, the expression of miR-7-5p, miR-183-5p, miR203a-3p and miR-215 was either increased or decreased by transfection of the cells with the respective miRNA-mimic or inhibitor (antagomir). Transient transfection was performed with Lipofectamine 2000 (Thermo Fisher Scientific, Waltham, MA, USA) in accordance to the manufacturer's instructions. In brief, AT1 and Walker-256 cells (0.5–0.7⋅10^6^ cells/ml) were incubated with lipofectamine and the respective miRNA mimic or antagomir (miRCURY LNA, Qiagen, Hilden Germany) for 24 h. The list of miRNA sequences used for transfection is shown in Suppl. Table S1. Transfection with unspecific miRNA sequences served as controls (Qiagen). Mimics were used at a final concentration of 1.7 nM and antagomirs at 16.7 nM. After 24 h, the lipofectamine containing medium was replaced with media with different pH, in which the cells were incubated for another 24 h.

For *in vivo* experiments different transfection procedures were used. For experiments with miRNA mimics the cells were transfected with the respective miRNA mimic and afterwards these cells were implanted as described above by subcutaneous injection. Even though the transfection using lipofectamine and the pure mimic is only transient, the intratumoral miRNA expression was 64-times higher 8 days after tumor cell implantation (Suppl. Fig. S1A). For inhibitor experiments untransfected tumor cells were implanted. When the tumors reached the desired volume, a small amount (20 µl) of invivofectamine + respective miRNA inhibitor was injected intratumorally (under isoflurane anesthesia). The RNA strands are taken up into the cells and modulate the miRNA expression accordingly for at least 48 h (Suppl. Fig. S1B).

### mRNA expression

For mRNA expression analyses total RNA was isolated from cells or tumor homogenates using TRIzol according to the manufacturer's instructions. From previous experiments analyzing the impact of acidotic pH on gene expression [17] 13 genes (*Brip1, Clspn, Crem, Dnajc25, Ercc6l, Fstl1, Gls2, Ikbke, Il6r, Per3, Rif1, Tlr5, Txnip*) were identified, which were consistently regulated by low pH in both cell lines *in vitro* or *in vivo* and which have been described in literature to play a relevant rule in the malignant progression of tumors. For qPCR validation 1 µg RNA was subjected to reverse transcription with SuperScript II reverse transcriptase (Thermo Fisher Scientific) and analyzed by qPCR using the Platinum SYBR Green qPCR Supermix (Thermo Fisher Scientific). The obtained data were normalized against *18S* or *Hprt1*, which are suitable housekeeping genes for studying tumor acidosis [20], and were related to the respective control. Suppl. Table S2 shows the primers used.

### Tumor cell migration

The migratory speed of transfected (miRNA mimic or inhibitor) AT1 tumor cells was determined after 24 h incubation at pH 7.4 or 6.6. For time lapse microscopy 6 × 10^5^ cells were grown in 35 mm-Petri dishes, incubated with the buffers at different pH and transferred to an incubation chamber (stage Top Incubator INU-KI-F1; Tokai Hit) of a Keyence BZ-8100E fluorescence microscope (Keyence, Osaka Japan). Cell migration was measured over a time interval of 100 min with imaging every 5 min. Single cells were tracked in this series of 20 images and the averaged migratory speed (in µm/min) as well as the covered distance (in µm) was determined. For the calculations ImageJ software (ibidi Chemotaxis and Migration Tool, Gräfelfing, Germany) was used.

### Cell adhesion

Cell adhesion was measured by continuous impedance measurements of monolayer cells (xCELLigence DP; OLS OMNI Life Science, Bremen, Germany) in accordance to the manufacturer's instructions. First, it was tested whether cells lose their adherence if they are exposed to low pH. Therefore, cells were plated on 16-well plates for 48 h to establish a tight contact between cells and plate surface. Thereafter, medium was changed to pH 7.4 or pH 6.6 and impedance was followed for 48 h. In the second series it was tested whether priming the cells at low pH for 24 h will affect the ability to adhere on the surface. Therefore, cells were pre-incubated at pH 6.6 or 7.4 in petri dishes for 24 h. Subsequently, cells were mechanically detached and the cell suspensions were then transferred to 16-well plates in which the impedance was measured during the next 48 h.

### Cell cycle distribution and proliferation

For analysis of DNA content and the fraction of actively DNA-synthesizing cells, cells were incubated with 5 µM BrdU (Bromodeoxyuridine) for 1h (*in vitro*) or 2h (*in vivo*). Cells were then fixed with 70% ethanol and stained with anti-BrdU-antibody or isotype control (BD Biosciences, San Jose, CA, USA) and secondary anti-mouse-FITC-antibody (1:100) (Rockland, Limerick, PA, USA). Additionally, cells were stained for 10 min with 50 µg/ml propidium iodide+RNase to measure cell cycle distribution. For analyses of tumors, BrdU was dissolved in PBS and injected i.p. (150 mg/kg body wt). After 120 min, tumors were excised and mechanically disintegrated into a single cell suspension and treated as stated above.

### Apoptosis and necrosis

Caspase-dependent apoptosis was assessed by measuring the activity of the effector caspase-3 as described previously. In brief, cells were lysed, centrifugated and the supernatant was incubated with DEVD-AFC. The fluorescence of the cleaved dye 7-amino-4-trifluoromethylcoumarin (AFC) was measured in a multiwell reader (Infinite, Tecan, Berlin, Germany). Protein content was determined with Pierce BCA protein assay (Thermo scientific, Waltham, MA, USA) using bovine serum albumin as standard. For measurements in tumor samples small tissue specimens were minced before lysis. Necrosis in cultured cells was measured by LDH release. LDH activity in media and in cell lysates was measured using standard protocol adapted to lower scale (200 µl).

### Statistical analysis

Results are expressed as means±SEM. Differences between groups were assessed by the two-tailed t-test for paired and unpaired samples. The significance level was set at α=5% for all comparisons.

## Results

### The role of acidosis-regulated miRNAs in gene expression

In order to analyze whether acidosis-regulated miRNAs (miR-7, miR-183, miR-203, miR-215) affect gene expression, tumor cells were transfected either with mimics or inhibitors (antagomirs) of the respective miRNA before they were exposed to different pH values. Since miR-183, 203 and 215 were down-regulated under acidosis, the cells were transfected with the inhibitors at pH 7.4 and with mimics at pH 6.6. For miR-7 (which was up-regulated by acidosis) transfection was performed with the mimics at pH 7.4 and antagomirs at pH 6.6.

**Fig. 1:**
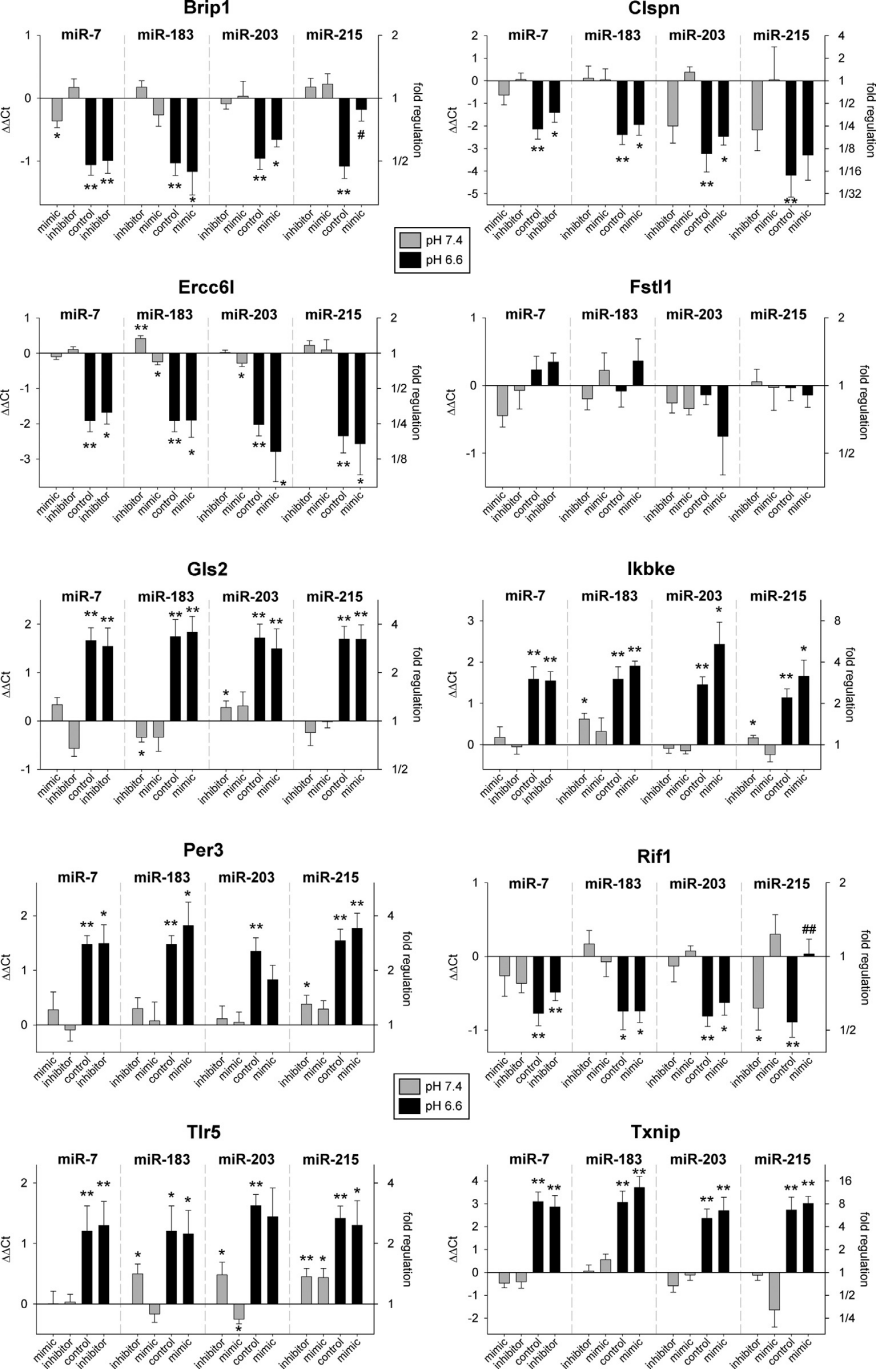
mRNA expression of tumor-associated genes in AT1 prostate carcinoma cells after 24 h at pH 7.4 or 6.6 in combination with overexpression (mimic) or inhibition of pH-dependent miRNAs. Mean ± SEM, *n*=3–20, (*) *p* < 0.05, (**) *p* < 0.01 *vs*. pH 7.4 control; (#) *p* < 0.05, (##) *p* < 0.01 *vs*. pH 6.6 control (without transfection at the respective pH).

**Fig. 2:**
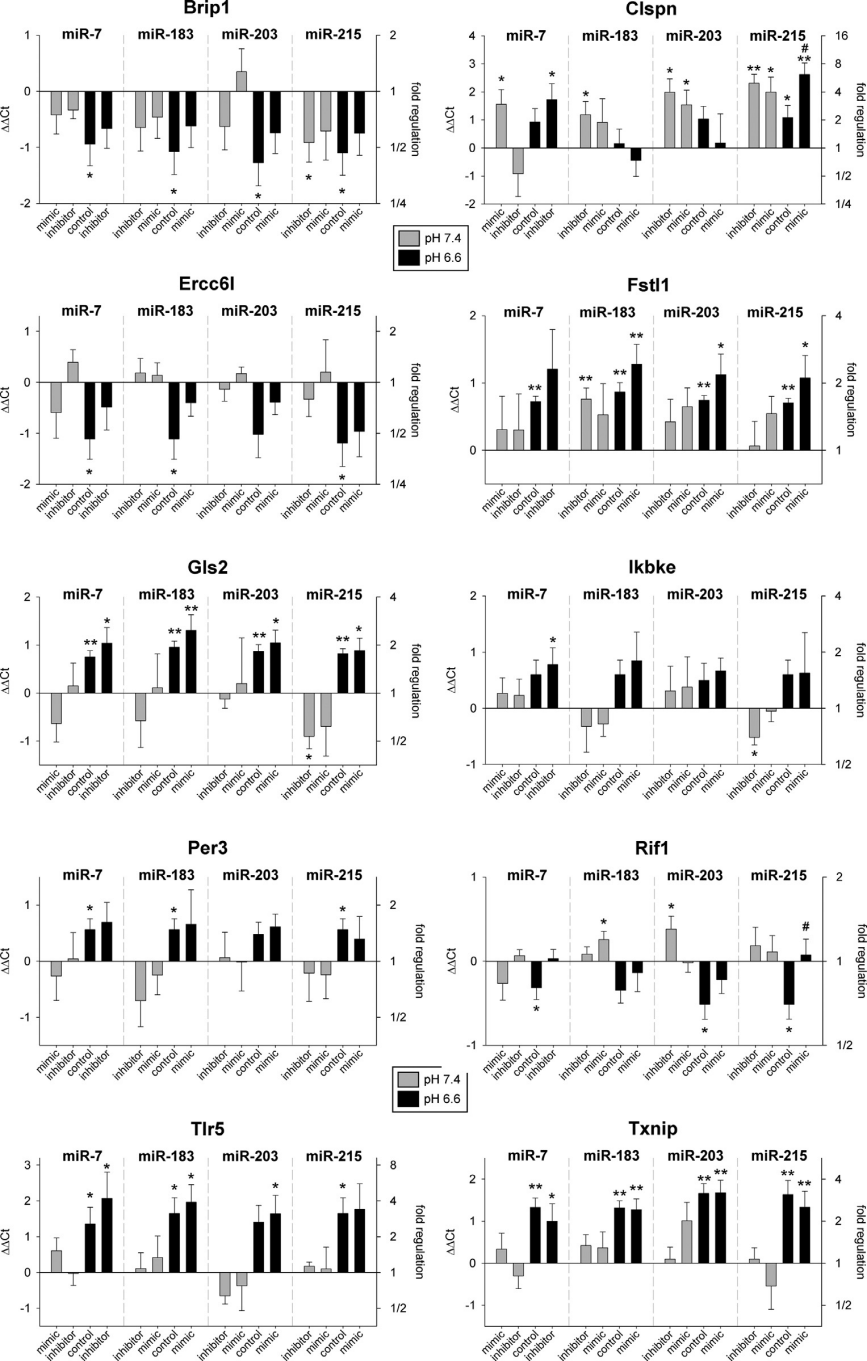
mRNA expression of tumor-associated genes in Walker-256 mammary carcinoma cells after 24 h at pH 7.4 or 6.6 in combination with overexpression (mimic) or inhibition of pH-dependent miRNAs. Mean ± SEM, *n*=3–13, (*) *p* < 0.05, (**) *p* < 0.01 *vs*. pH 7.4 control; (#) *p* < 0.05 *vs*. pH 6.6 control.

Figs. 1 and 2 and Suppl. S2 show the effects of miRNA-transfection in AT1 and Walker-256 cells. In the majority of genes, the acidotic extracellular pH had a significant impact on mRNA expression compared to control conditions at pH 7.4. However, in most cases the additional transfection did not influence the expression markedly (e.g., Gls2 or Txnip in Fig. 1). Only in a few genes, miRNA-expression had a direct impact, for instance miR-215 in AT1 (Fig. 1). At control pH (7.4) transfection with the inhibitor reduced Rif1 expression (comparable to acidotic control conditions at which the miR-215 expression was reduced). Under acidic conditions transfection with miR-215 mimic increased Rif1 expression to the non-acidotic control level. In Walker-256 cells similar effects of miR-215 on Rif1 expression were seen (Fig. 2) where acidosis reduced the expression whereas simultaneous transfection with miR-215 mimic led to almost control level at pH 7.4. In AT1 cells miR-215 also had an impact on Brip1 (Fig. 1). Here, miR-215 overexpression changed Brip1 expression back to the control level. However, in this case inhibition of miR-215 at pH 7.4 did not reduce the Brip1 expression. In Walker-256 cells, inhibition of miR-215 reduced the Brip1 expression at pH 7.4 (Fig. 2). These results indicate that the acidosis-induced change of Rif1 (and possibly also Brip1) expression could be mediated by the pH-dependent miR-215.

**Fig. 3:**
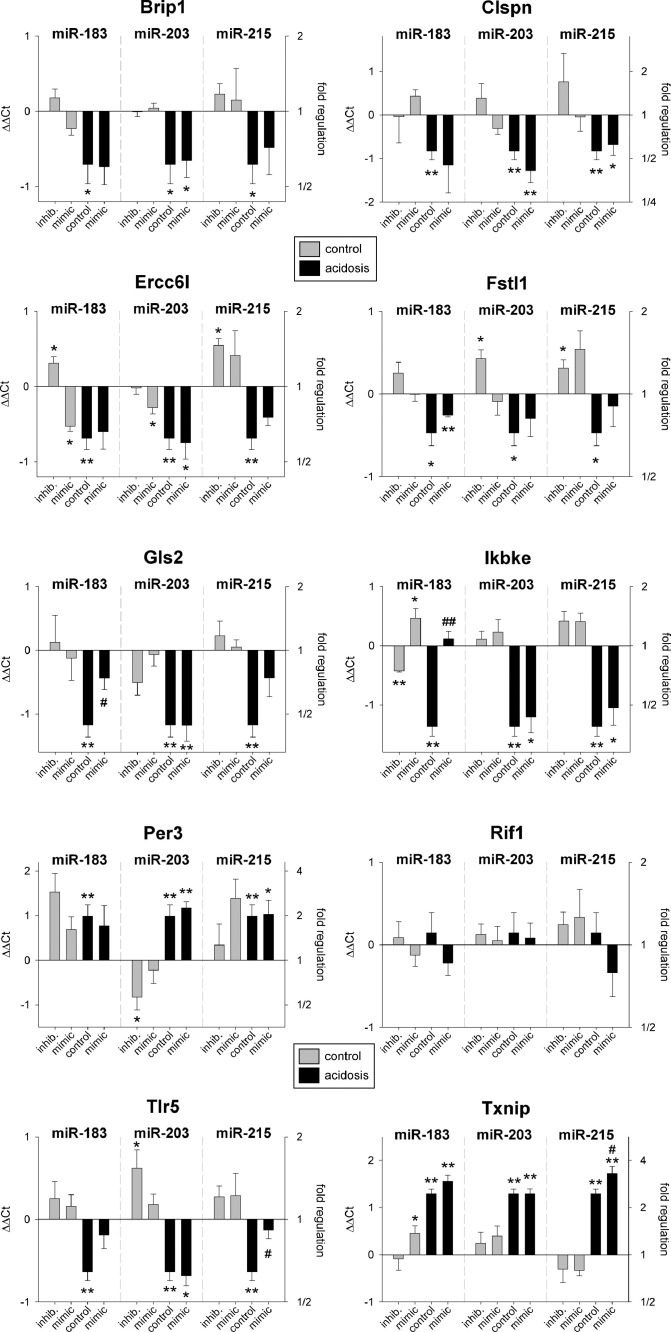
mRNA expression of tumor-associated genes in AT1 tumors after 24 h under control or acidotic conditions in combination with overexpression (mimic) or inhibition of pH-dependent miRNAs. Mean ± SEM, *n*=3–9, (*) *p* < 0.05, (**) *p* < 0.01 *vs*. pH 7.4 control; (#) *p* < 0.05, (##) *p* < 0.01 *vs*. pH 6.6 control.

In solid experimental tumors *in vivo* different expression patterns were found. In AT1 tumors neither Rif1 nor Brip1 expression was modulated significantly by miR-215 (Figs. 3, Suppl. S3), instead Gls2 was regulated by miR-215 and miR-183. Overexpression of both miRNAs reversed the acidosis-induced decrease in Gls2 expression. Surprisingly, overexpression of the miR-215 induced a further increase of the Txnip expression in AT1 tumors rather intensifying the acidosis effect than counteracting it (Fig. 3). Additionally, miR-183 had a regulating effect on Ikbke and Tlr5 expression. For both genes, overexpression of miR-183 under acidic conditions counteracted the acidosis induced down-regulation and led to an expression level similar to control tumors at normal pH. These results indicate that in solid tumors besides miR-215 also miR-183 may be involved in the acidosis-induced changes of gene expression.

Taking *in vitro* and *in vivo* results together, it can be concluded that the expression of several genes (Brip1, Gls2, Rif1, Ikbke, Tlr5, Txnip) under acidic conditions might be (at least partially) mediated by different pH-dependent miRNAs. The overexpression or inhibition of these miRNAs was able to simulate or counteract the acidosis-induced changes. Here, especially the miR-183 and miR-215, which are both down-regulated at low pH, seem to play a relevant role.

### Acidosis-regulated miRNAs regulating functional parameters

Since genes, which are relevant for proliferation, cell death or migration, are regulated by extracellular acidosis and miRNAs, it was tested whether changes in the expression of pH-dependent miRNAs have functional consequences for tumor growth, cell cycle distribution, apoptosis, necrosis, migration as well as for tumor cell adhesion *in vitro* and/or *in vivo*.

**Fig. 4:**
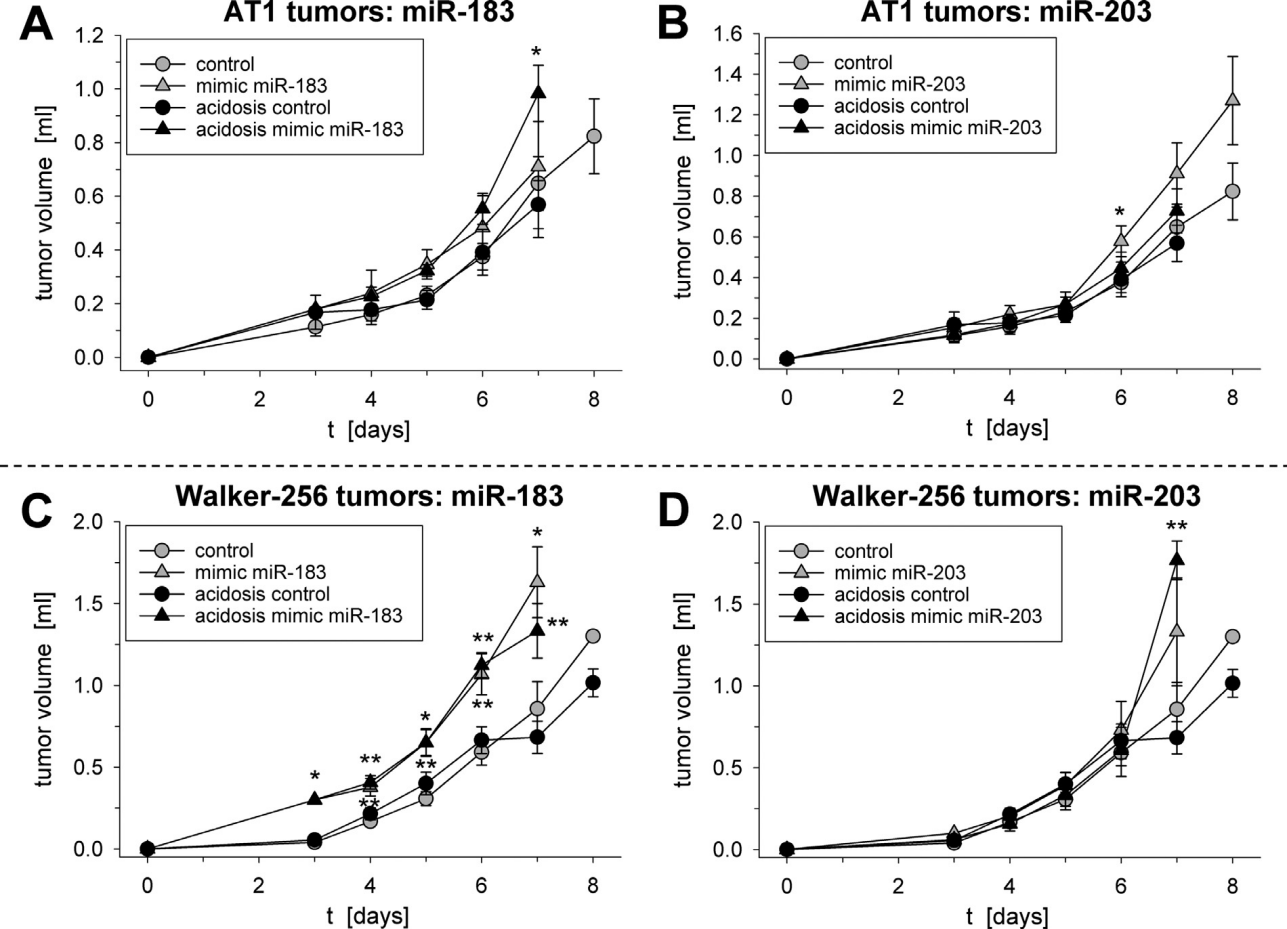
Tumor growth of AT1 (**A**+**B**) and Walker-256 tumors (**C**+**D**) under acidic conditions and with transfection of the tumor cells with mimics of the miR-183 (**A**+**C**) or miR-203 (**B**+**D**). Mean ± SEM, *n* = 3–17; (*) *p* < 0.05, (**) *p* < 0.01 *vs.* control.

**Fig. 5:**
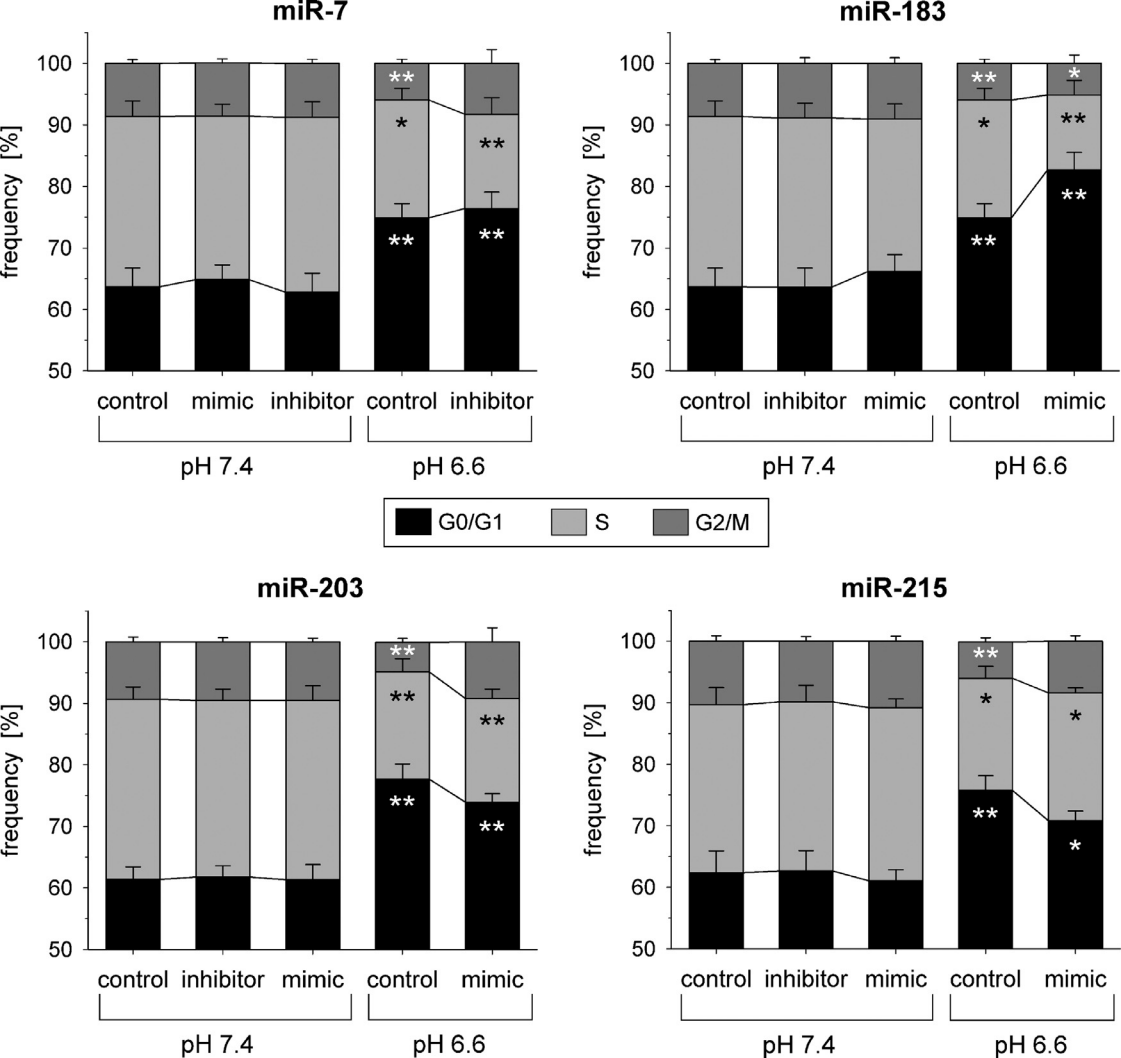
Cell cycle distribution of AT1 prostate carcinoma cells after 24 h at pH 7.4 or 6.6 in combination with overexpression (mimic) or inhibition of pH-dependent miRNAs. Mean ± SEM, *n*=5–14, (*) *p* < 0.05, (**) *p* < 0.01 *vs*. pH 7.4 control.

**Fig. 6:**
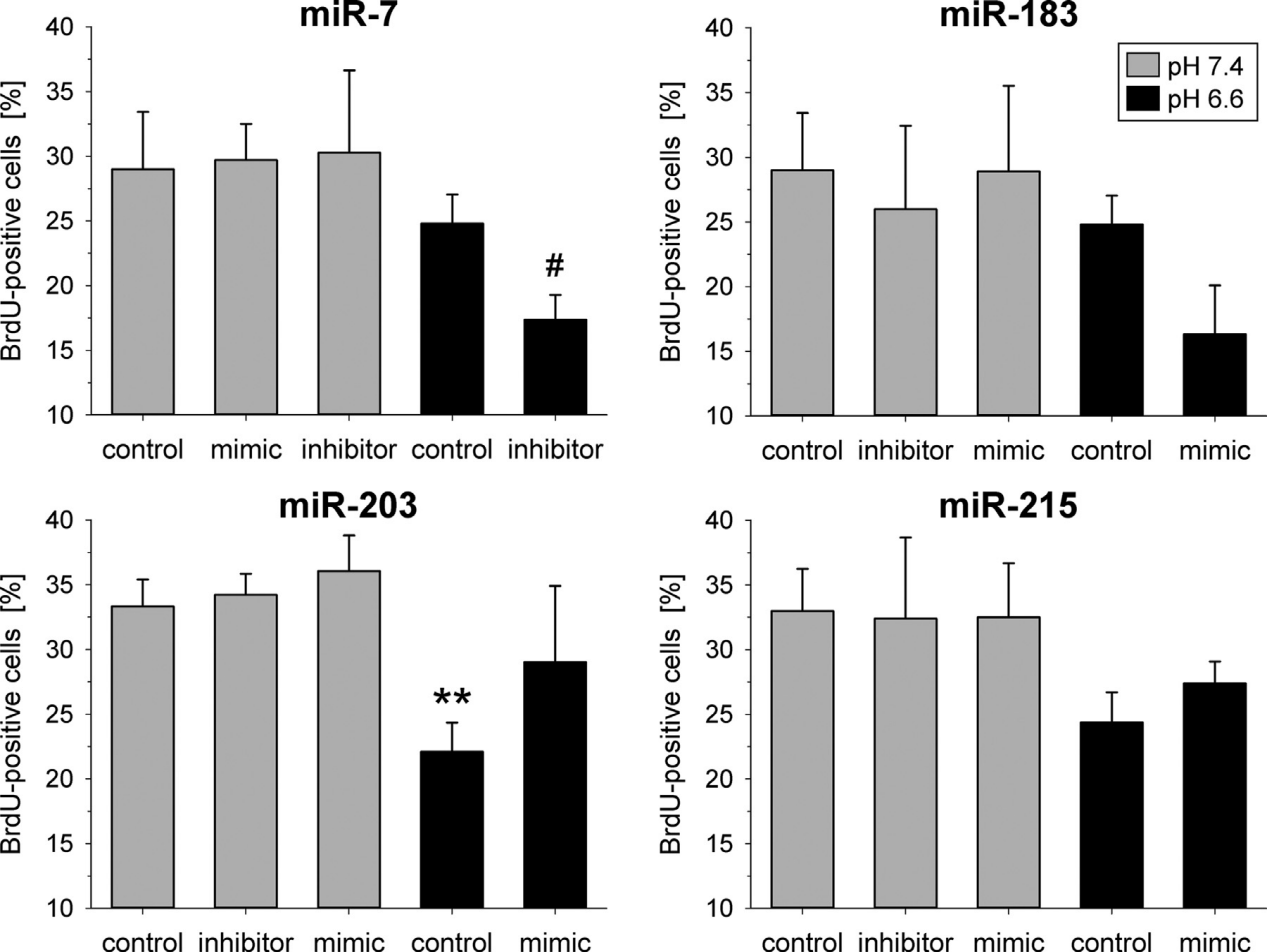
Fraction of proliferating AT1 cells (BrdU uptake) after 24 h at pH 7.4 or 6.6 in combination with overexpression (mimic) or inhibition of pH-dependent miRNAs. Mean ± SEM, *n*=5–14, (**) *p* < 0.01 *vs*. pH 7.4 control; (#) *p* < 0.05 *vs*. pH 6.6 control.

Under *in vivo* conditions the growth rate of tumors was almost unaffected from the extracellular pH. If the tumors were transfected with miR-183 or miR-203 (most pronounced in Walker-256 tumors after transfection with the miR-183), tumor growth was significantly faster (Fig. 4) whereas miR-215 had practically no effect (data not shown). Interestingly, in AT1 cells miR-183 overexpression led at low pH to a stronger G0/G1 arrest (Fig. 5) and a reduced BrdU incorporation (Fig. 6) indicating diminished cell proliferation. However, in Walker-256 cells all miRNAs had almost no impact on cell-cycle distribution (Suppl. Fig. S4) and although miR-7 and 215 increased proliferation markedly, the effect was not statistically significant (Suppl. Fig. S5). As a second measure of proliferation the number of cells after 24 h (expressed by the protein content in the petri dish) was analyzed. In both cell lines acidosis reduced the cell number (protein concentration) by about 50% (Suppl. Fig. S6), but only overexpression of miR-203 in AT1 cells (under acidic conditions) increased the cell division (Suppl. Fig. S6A). These data from cultured AT1 cells are in accordance with the results from the tumor growth *in vivo* (Fig. 4).

**Fig 7:**
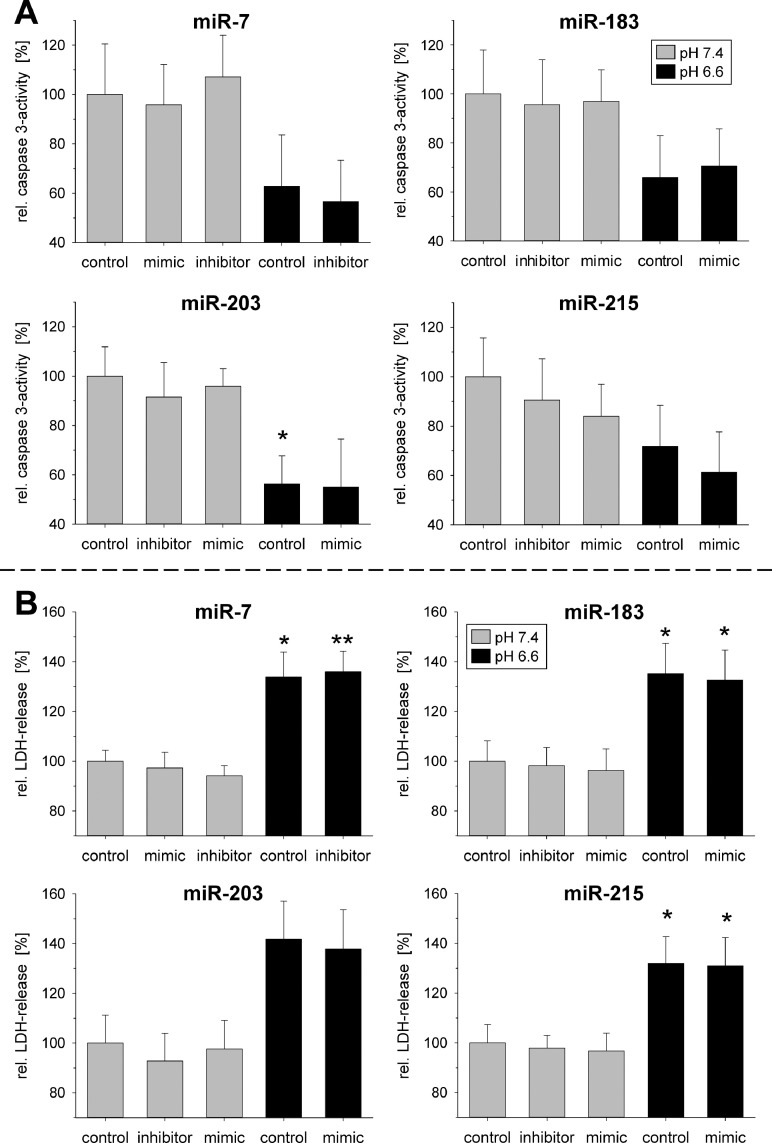
(**A**) Apoptosis (caspase 3-activity) and (**B**) necrosis (LDH release) of Walker-256 cells after 24 h at pH 7.4 or 6.6 in combination with overexpression (mimic) or inhibition of pH-dependent miRNAs. Mean ± SEM, *n*=5–9, (*) *p* < 0.05, (**) *p* < 0.01 *vs*. pH 7.4 control.

The faster tumor growth *in vivo* of tumors transfected with miR-183 or 203 may not only be the result of an impaired cell proliferation but also of differences in apoptotic or necrotic cell death. In Walker-256 cells the extracellular acidosis *per se* strongly reduced caspase-3 activity (Fig. 7A) and significantly increased necrotic cell death (Fig. 7B). However, none of the miRNAs further modulated apoptosis or necrosis (Fig. 7). In AT1 cells transfection with miR-183 or miR-203 under acidic condition induced a slightly lower caspase-3 activity (Suppl. Fig. S7A) but had almost no impact on necrosis at low pH (Suppl. Fig. S7B). At pH 7.4 miR-183 overexpression led to an increase in necrosis.

**Fig. 8:**
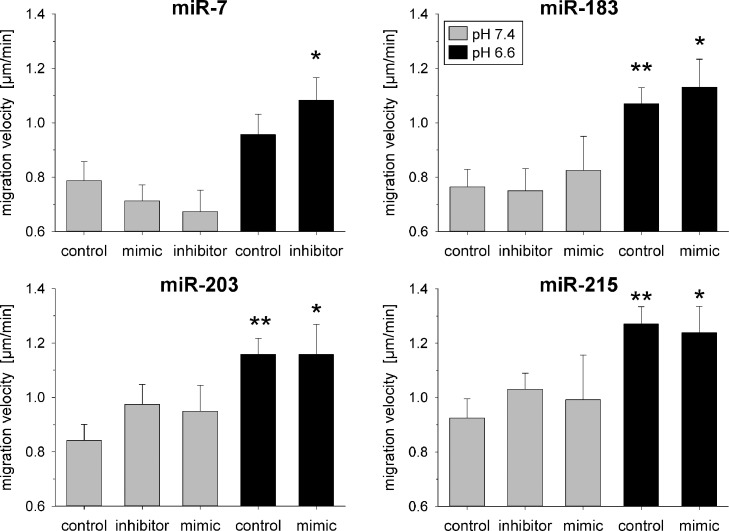
Migration velocity of AT1 cells after 24 h at pH 7.4 or 6.6 in combination with overexpression (mimic) or inhibition of pH-dependent miRNAs. Mean ± SEM, *n*=5–14, (*) *p* < 0.05, (**) *p* < 0.01 *vs*. pH 7.4 control.

The migratory potential of tumor cells was assessed from the migration velocity and from the covered distance from the starting point. Under acidic conditions (without transfection) the migratory speed (Fig. 8) as well as the covered distance (Suppl. Fig. S8) of AT1 cells were significantly increased indicating a higher mobility of tumor cells at low pH. If the cells were additionally transfected any of the pH-dependent miRNAs neither the migratory speed nor the covered distance was significantly altered (Figs. 8 and Suppl. S8).

**Fig. 9:**
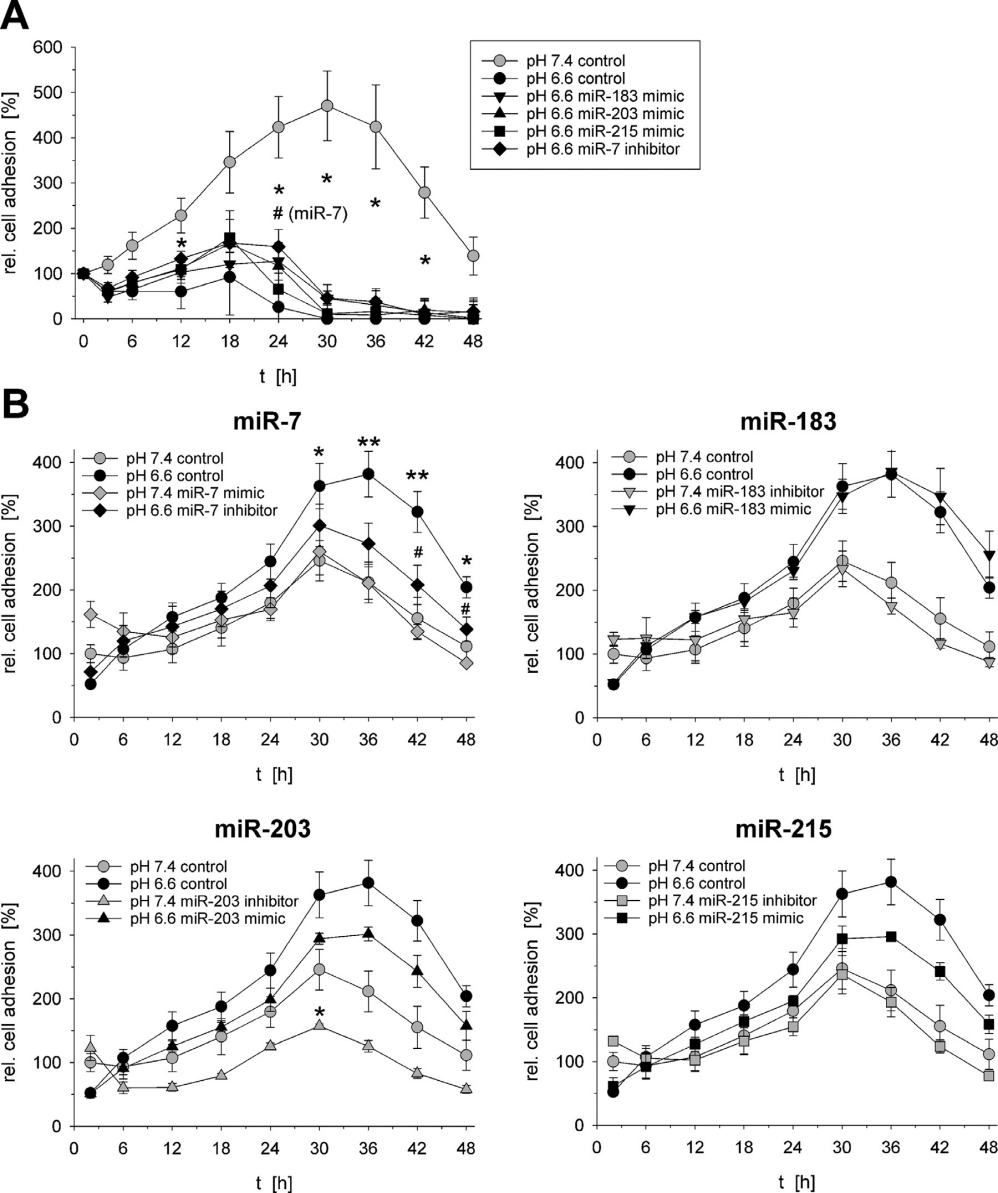
Adhesion of AT1 cells after 24 h at pH 7.4 or 6.6 in combination with overexpression (mimic) or inhibition of pH-dependent miRNAs. (**A**) At t=0 h the medium of already adherent cells was changed to pH 6.6 ("direct incubation"). (**B**) Cells were pre-incubated at pH 7.4 or pH 6.6 for 24 h then mechanically detached and again plated at pH 7.4 (*t*=0 h; "pre-incubation"). Mean ± SEM, *n*=2–5, (*) *p* < 0.05, (**) *p* < 0.01 *vs*. pH 7.4 control; (#) *p* < 0.05 *vs*. pH 6.6 control.

In addition, the adhesion of tumor cells was modulated by extracellular pH and miRNA expression. In order to simulate different processes of metastasis, two different experimental settings were used. To mimic the detachment of single cells from an acidic tumor, the medium of already adherent cells was changed to pH 6.6 ("direct incubation") and the adherence was analyzed during the next 48 h by impedance measurements. To simulate the invasion of circulating tumor cells, which originate from an acidic tumor, into a new host tissue, the cells were pre-incubated at pH 6.6 for 24 h, then mechanically detached and the following process of adherence (now at pH 7.4) was measured ("pre-incubation"). During the "direct incubation" experiment low pH induced a significant reduction of cell adherence (Fig. 9A) at which the simultaneous transfection of the cells with miR-7 inhibitor slightly diminished this reduction of adhesion. For the other pH-dependent miRNAs no consistent effect was seen. In the "pre-incubation" experiments acidosis induced a stronger adherence (Fig. 9B). This effect was antagonized by transfecting the cells with the miR-7 inhibitor. Also the transfection with miR-203 or miR-215 mimic reduced adhesion (compared to acidotic control; Fig. 9B). These results reveal that especially miR-7 seems to play a relevant role for changes in tumor cell adhesion.

## Discussion

The aim of the study was to test whether the impact of extracellular acidosis on gene expression and functional properties of tumor cells is mediated by pH-sensitive miRNAs (miR-7, miR-183, miR-203, miR-215). Therefore, the cells were transfected with either mimics or inhibitors of these miRNAs at different pH *in vitro* and *in vivo*. Numerous genes (such as Brip1, Clspn, Gls2, Per1, Rif1, Tlr5, Txnip) were regulated by an acidic extracellular pH (Figs. 1, 2), which has already been described in previous studies [17]. However, in most of the cases an up- or down-regulation of the miRNA expression had almost no impact on gene expression. In AT1 cells (Fig. 1) only the expression of Brip1 and Rif1 showed a significant change by miR-215. In both cases, overexpression of miR-215 at low pH (miR-215 expression was reduced by acidosis) was able to return the gene expression to the control level at pH 7.4. In Walker-256 cells (Fig. 2) also Rif1 expression was sensitive to miR-215 expression, at which again the miRNA overexpression at low pH led to Rif1 expression comparable to control conditions (pH 7.4). In Walker-256 cells miR-215 overexpression also affected Clspn expression but in this case gene expression during acidosis was further increased. From all genes tested only Rif1 seems to be cell line-independently regulated by the pH-sensitive miR-215. Under acidic conditions, Rif1 expression was reduced and overexpression of miR-215 counteracted this down-regulation. These results reveal that miR-215 seems to control the expression of Rif1 under acidic conditions. However, the impact of this interaction for tumor progression is presently not conclusive. On the one hand, miR-215 has been described as a tumor suppressor [21], [22], [23], [24] and thus a reduced expression should be indicative for more progressive tumor growth. On the other hand, Rif1 is a regulatory factor of DNA damage response [25] and by this down-regulation has been described to increase the sensitivity of tumors to platinum-based chemotherapy or irradiation [26,27]. Additionally, Rif1 may directly foster tumor cell proliferation [28,29].

In solid tumors neither acidosis *per se* nor miR-215 had an impact on Rif1 expression. In contrast, some other genes were found to depend on miR-215 expression (and acidosis), such as Tlr5, Txnip or Gls2 (Fig. 3). These genes were not regulated in isolated tumor cells (Fig. 1). In tumors miR-183 was found to modulate the Gls2 expression under acidic condition significantly, which was also not seen in cell culture. The difference between cell culture and tumors may result from the fact that in solid malignancies not only tumor cells are present but also different normal cells such as fibroblasts, endothelial cells or macrophages. Additionally, communication between tumor and normal cells, either by direct cell-cell contacts or by secreted cytokines (which has also been shown to be acidosis-induced [30]) may contribute to differences in gene expression *in vitro* and *in vivo*.

The analysis of functional parameters (proliferation, cell death, migration, adhesion) revealed first, that two of the pH-dependent miRNAs have a direct impact on tumor growth. In both tumor lines overexpression of miR-183 led to faster tumor growth which was even more pronounced in Walker-256 tumors (Fig. 4A–C). The impact of miR-183 is in accordance to previous studies demonstrating a positive correlation of miR-183 expression and tumor progression [31], [32], [33]. From these data it can be concluded that the acidosis-induced down-regulation of miR-183 may slow down tumor growth. Although in the present study, no significant difference in the volume of acidic and control tumors was seen (Fig. 4), it has to be taken into account that the artificial tumor acidification took place first after day 6 of the tumor growth experiment and lasted no longer than 24 h.

The increased tumor growth can result from either an increased proliferation or a reduced cell death. Proliferation and apoptotic or necrotic cell death were differentially affected by acidosis and/or miRNA expression in both cell lines. In AT1 cells preferentially proliferation was modulated, whereas in Walker-256 cells primarily apoptosis and necrosis were affected. In AT1 cells acidosis led to a significant G1 arrest (Fig. 5) and a marked reduction in the fraction of proliferating cells (BrdU incorporation; Fig. 6) which has been described by others [34] and may be associated with pH-dependent changes in gene expression as for instance for Gls2 (Fig. 1) [35]. The pH effect could be modulated by the overexpression of miR-183, intensifying the G1 arrest, whereas miR-203 and miR-215 reduced it (but not statistically significant). These changes were also reflected by trend in the BrdU uptake (Figs. 5, Suppl. S5). Even though a miR-203-induced G1 arrest was already described previously [36,37], the effect only occurred under acidic conditions but not at pH 7.4. In AT1 cells miR-203 expression might already be strongly induced under control conditions, possibly explaining that a further increase by miR-203 mimic transfection had no impact on the cell cycle. Overexpression of miR-215 under acidic conditions led to a reduction of cells in the G0/G1 phase and an increase of those in the G2/M phase of the cell cycle. A miR-215 mediated G2/M arrest has been described by others [22,38], but similar to miR-203, the impact of miR-215 overexpression was not seen at pH 7.4. Taking these results together, it may be possible that the changes in proliferation were at least partially mediated by changes of the miRNAs, but seem to be cell line-specific. The apoptotic and necrotic cells death was also acidosis dependent, but this effect was fundamentally different in both cell lines. In Walker-256 cells low pH induced a reduction of apoptosis and a strong increase in necrosis (Fig. 7) whereas in AT1 cells acidosis had only a negligible effect. In both cell lines, cell death was not altered by changes in microRNA expression concluding that pH-dependent miRNAs play no significant role in apoptosis or necrosis.

In the present study additionally the impact of acidosis and pH-dependent miRNAs on migration and adhesion was analyzed. Acidosis *per se*, as has already been described previously [6,17], increased the migration speed (Fig. 8) and the covered distance in the migration experiment (Suppl. Fig. S8). Other authors have shown that miR-7 overexpression [39], [40], [41] as well as miR-183 down-regulation [42], [43], [44] lead to an increase of tumor cell migration. However, in the present experiments up- or down-regulation of any of these miRNAs showed a modulatory effect. For this reason, it can be concluded that the migration effects of the acidic environment are probably not mediated by pH-sensitive miRNAs.

Cell adhesion was also found to be not only affected by pH but also by several miRNAs. To investigate different steps of metastatic spread two different experiments were performed. To simulate the shedding of tumor cells from an acidic solid tumor mass, the detaching of the cells after switching to low pH was analyzed ("direct incubation") (Fig. 9A). Here acidosis induced a significant detachment, but none of the miRNAs had a marked impact on this process. The second experiments simulated the process of invasion of circulating tumor cells (originating from an acidic tumor) into a new host tissue with apre-incubation at low pH (Fig. 9B). Acidosis *per se* increased adhesion but this effect was also depending on miRNA expression. Overexpression of miR-203 and miR-215 as well as down-regulation of miR-7, in conjunction with low pH, reduced cell adhesion. For miR-203 and miR-215 it has been shown that overexpression may lead to reduced adhesion [45,46] which is in accordance with the present study. Since for miR-7 overexpression also a suppression of adhesion via modulation of the focal adhesion kinase (FAK) has been described [47]. The reason for the oppositional effect in the present study is unclear.

In conclusion, the present study analyzed whether pH-dependent miRNAs (miR-7, miR-183, miR-203, miR-215) may play a role in the signaling cascade in which the extracellular H^+^ concentration modulates gene expression and functional properties of tumor cells. The study revealed that many genes were affected by tumor acidosis but only for a few, a direct impact of acid-regulated miRNAs was found, illustratingBrip1, Clspn, and Rif1 as the most relevant ones. These effects were partly cell line dependent and *in vivo* additional genes such as Fstl, Tlr5, or Txnip were modulated by these miRNAs. From the functional properties analyzed only cell cycle distribution, apoptosis and cell adhesion were markedly influenced by the miRNAs studied. Since in this screening study most of the effects were cell line-specific and in most cases only single miRNAs showed effects on particular genes or cell functions, further studies are needed to identify specific signaling cascades, leading from extracellular pH via acidosis-dependent miRNAs to gene expression and finally to changes of specific tumor cell properties.

## CRediT authorship contribution statement

**Mandy Rauschner:** Conceptualization, Investigation, Formal analysis, Validation, Writing – original draft, Writing – review & editing. **Thea Hüsing:** Investigation, Formal analysis, Writing – review & editing. **Luisa Lange:** Investigation, Formal analysis, Writing – review & editing. **Kristin Jarosik:** Investigation, Formal analysis, Writing – review & editing. **Sarah Reime:** Investigation. **Anne Riemann:** Conceptualization, Formal analysis, Validation, Visualization, Writing – original draft, Writing – review & editing. **Oliver Thews:** Conceptualization, Formal analysis, Validation, Visualization, Funding acquisition, Writing – original draft, Writing – review & editing.

## Declaration of Competing Interest

The authors declare that they have no known competing financial interests or personal relationships that could have appeared to influence the work reported in this paper.

## Appendix Supplementary materials

Image, application 1
