# Supplementary material for: Role of acidosis-sensitive microRNAs in gene expression and functional parameters of tumors in vitro and in vivo
Source: Neoplasia. 2021 Nov 13;23(12):1275–88. doi: 10.1016/j.neo.2021.11.005 (PMC8605108; doi:10.1016/j.neo.2021.11.005)
Supplement: Supplementary file 1 [file mmc1.pdf]

Impact of acidosis-dependent microRNAs on gene expression and functional parameters of tumors *in vitro* and *in vivo*

Mandy Rauschner, Thea Hüsing, Luisa Lange, Kristin Jarosik, Sarah Reime, Anne Riemann, Oliver Thews

Institute of Physiology, University Halle, Magdeburger Str. 6, 06112 Halle (Saale), Germany

Supplementary Material

Table S1: miRNA sequences of mimics and antagomirs used for transient transfection.

**target miRNA**	**function**	**miRNA strand**
rno-miR-7-5p	mimic	5'-UGGAAGACUAGUGAUUUUGUUGU-3'
rno-miR-7-5p	inhibitor (antagomir)	5'-ACAACAAAATCACTAGTCTTCC-3'
rno-miR-183-5p	mimic	5'-UAUGGCACUGGUAGAAUUCACU-3'
rno-miR-183-5p	inhibitor (antagomir)	5'-AGTGAATTCTACCAGTGCCAT-3'
rno-miR-203a	mimic	5'- GUGAAAUGUUUAGGACCACUAG-3'
rno-miR-203a-3p	inhibitor (antagomir)	5'-TAGTGGTCCTAAACATTTCA-3'
rno-miR-215	mimic	5'- AUGACCUAUGAAUUGACAGAC-3'
rno-miR-215	inhibitor (antagomir)	5'-GTCTGTCAATTCATAGGTCAT-3'

Table S2: Primers used for quantitative PCR.

**target**	**forward primer**	**reverse primer**
Brip1	CTCAGGGGTCCCGATGACTA	CTCCCGAGGCTGACAAGTTC
Clspn	AGCCAAGTGGAGAAAGGAGC	GGAAGAAGAGTTACCTCCCGC
Crem	TCCGAGCTCCTACTACTGCT	TTTCATCAGCCTCAGCTCCC
Dnajc25	GTCAGCGTGTGTGCCATTTC	TAGACCCACCGGCAATACCA
Ercc6l	GTTTAGCGGAAGTGGGGACT	CCAACTGCTCCAAAGCTTCC
Fstl1	TGCCCTCATTGAACTGTCCG	ACAGGAACAGACACAGCGAT
Gls2	CCTTTTCCTTTAGATGTGGGGC	AAGCAGGTCACCAAGTCGAG
Ikbke	ACCTGTAACTCAGAAGCCCG	TCCTGCATGTGGAAGACCAG
Il6r	CCTATACCCCTGCCCACATTC	TCAGCGGTCCCAAGGGATAC
Per3	GAACAGGAAACAACCGCACC	CCACGGGCTTGAATCCTTCT
Rif1	CACTGCGCTAGAAATGGGGA	TGTCCCACTACGGAAGCCTA
Tlr5	CTGTCTGACCTCAAGCGTGT	GGGCCACCTCAAATACTGCT
Txnip	CCAGCCTACAGGTGAGAACG	GGCTGGGACGATCGAGAAAA

**Housekeeper**		
Hprt1	ACCAGTCAACGGGGGACATA	TTGGGGCTGTACTGCTTGAC
18S	CTGAGAAACGGCTACCACATC	CCCAAGATCCAACTACGAGC

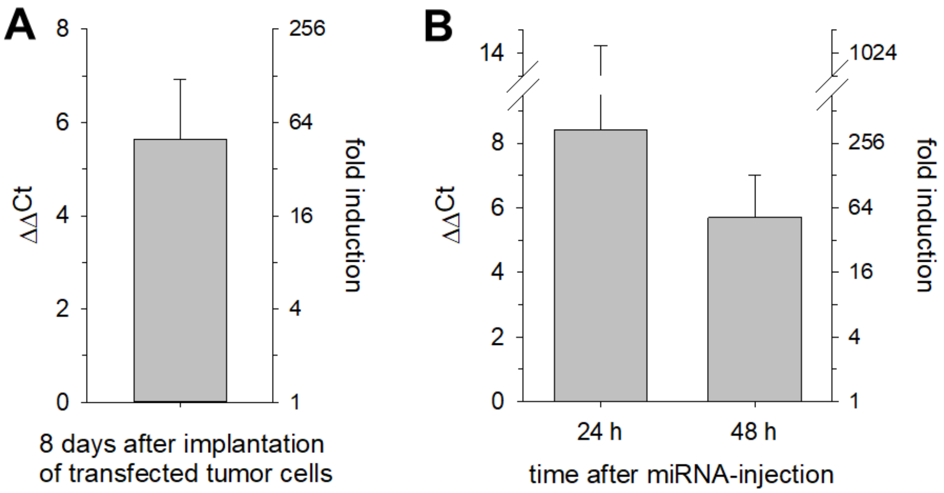

Fig. S1: Expression of miR-203 in AT-1 tumors *in vivo*. (**A**) 8 days after implantation of tumor cells transfected with miR-203 mimic. (**B**) 24 h and 48 h after a single intratumoral micro-injection (20 µl) lipofectamine with miR-203 mimic. Mean ± SEM, n=6-7.

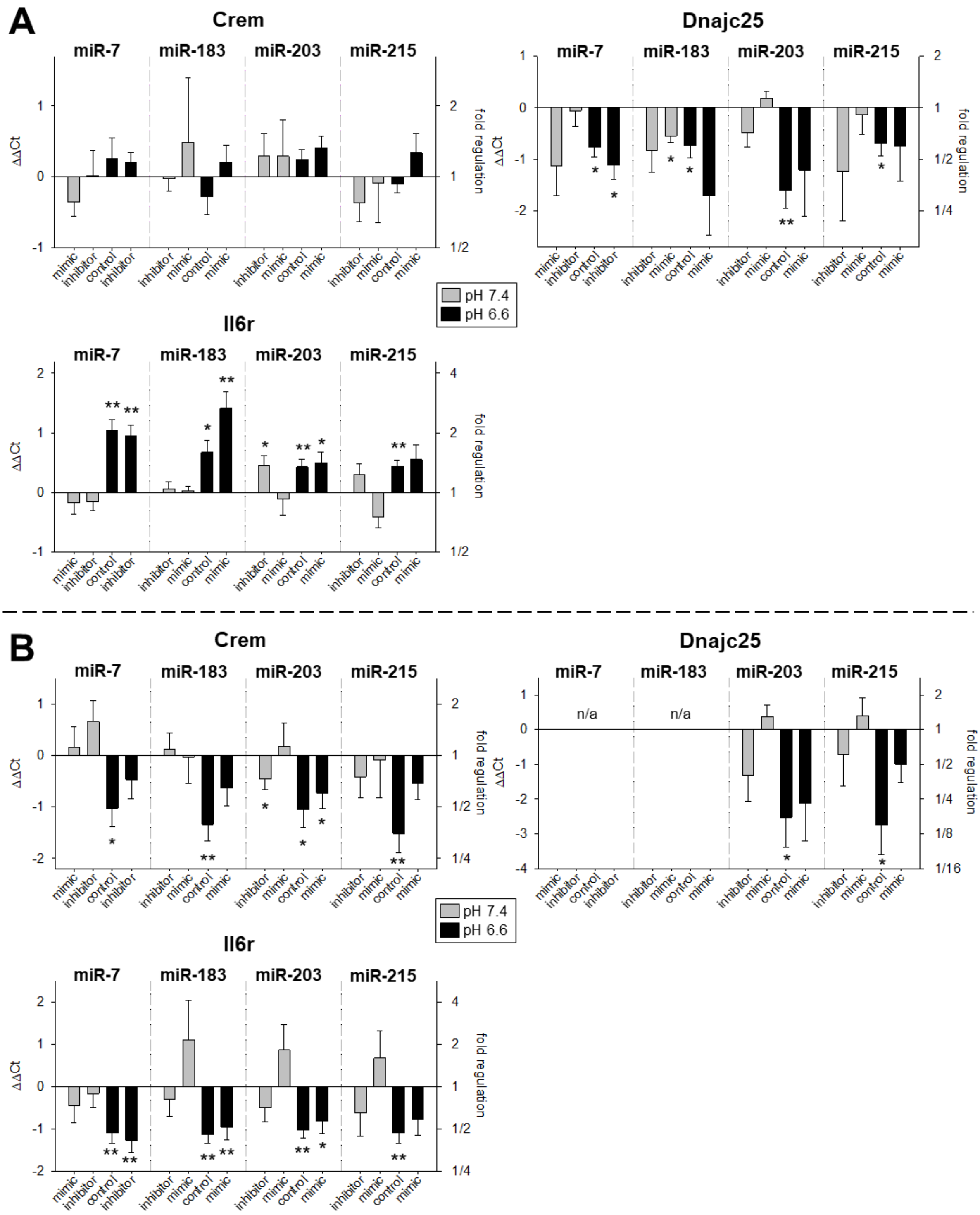

Fig. S2: mRNA expression of tumor-associated genes in (**A**) AT1 cells and (**B**) Walker-256 cells after 24 h at pH 7.4 or 6.6 in combination with overexpression (mimic) or inhibition of pH-dependent miRNAs. Mean ± SEM, n=2-15, (*) $p<0.05$, (**) $p<0.01$ vs. pH 7.4 control (n/a: not analyzed).

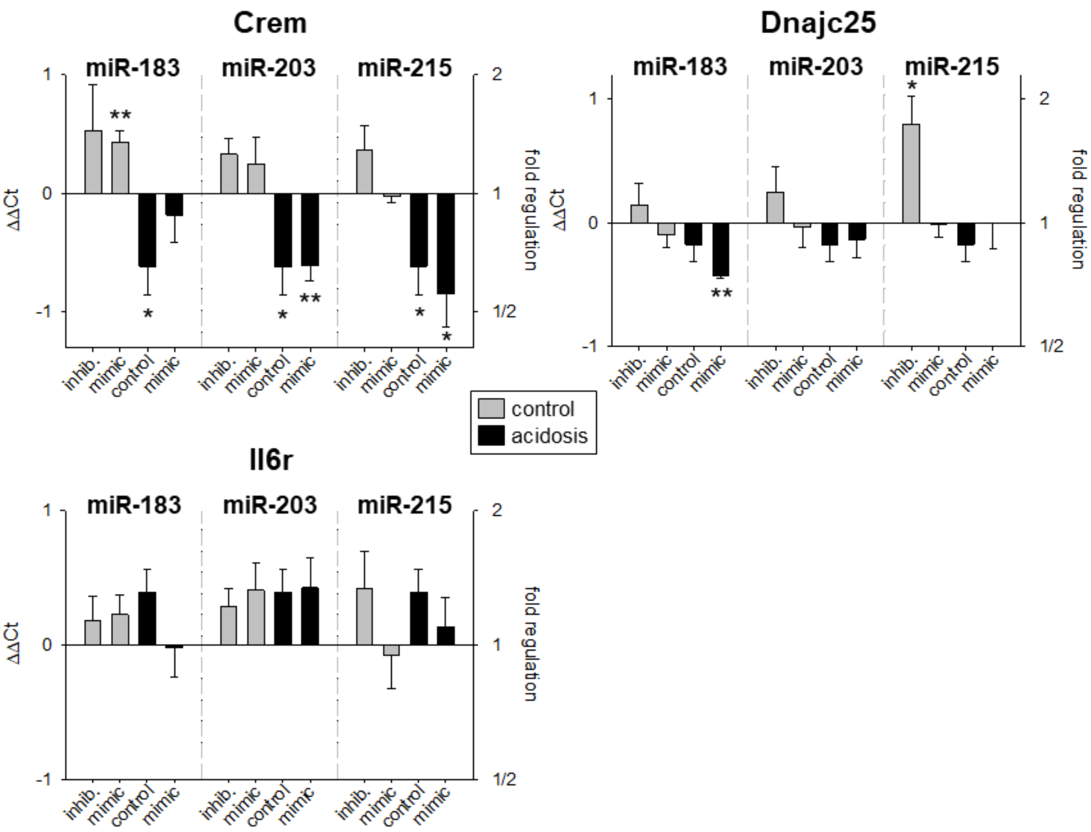

Fig. S3: mRNA expression of tumor-associated genes in AT1 tumors after 24 h under control or acidotic conditions in combination with overexpression (mimic) or inhibition of pH-dependent miRNAs. Mean ± SEM, n=3-8, (*) p<0.05, (**) p<0.01 vs. pH 7.4 control.

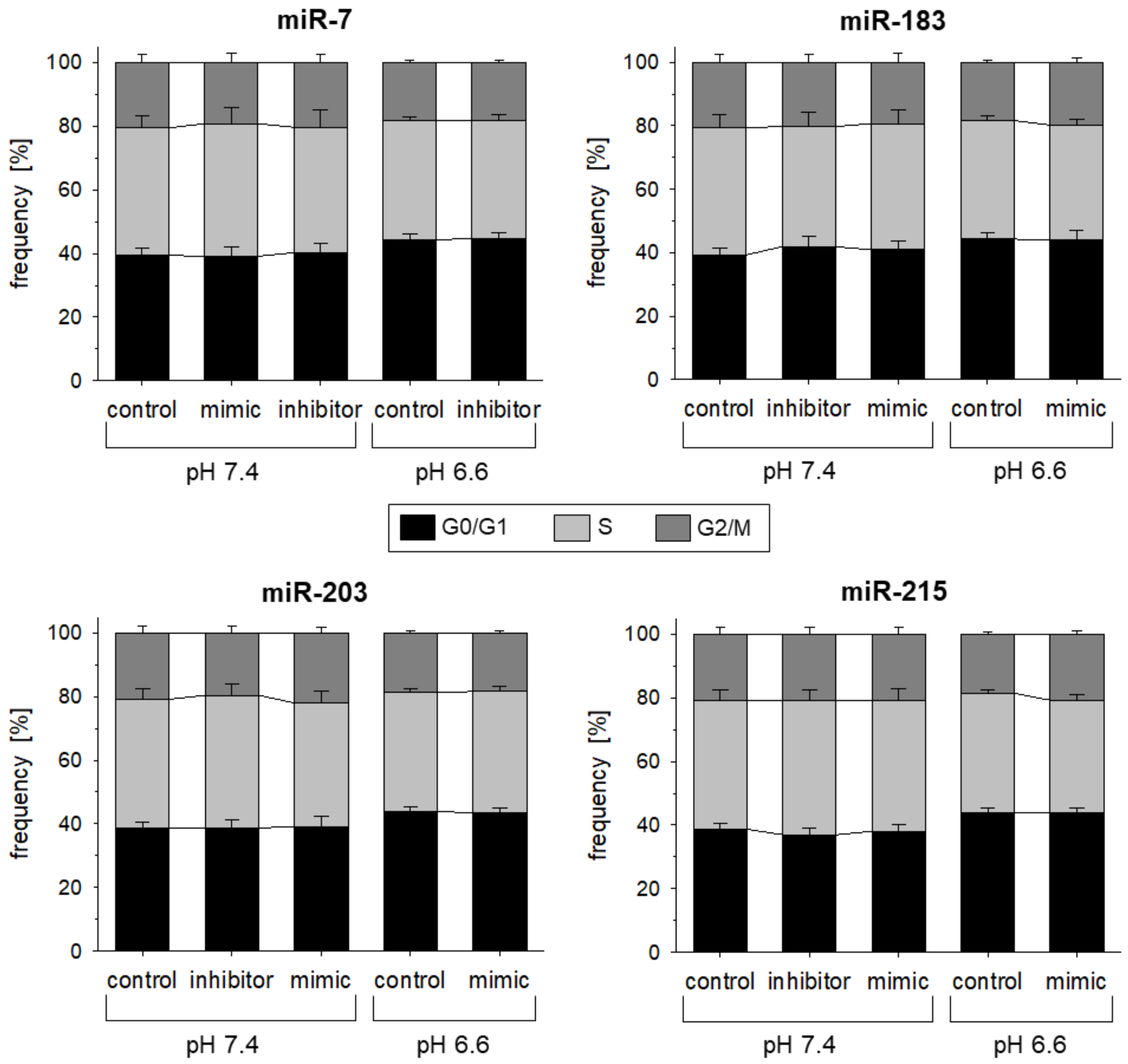

Fig. S4: Cell cycle distribution of Walker-256 mammary carcinoma cells after 24 h at pH 7.4 or 6.6 in combination with overexpression (mimic) or inhibition of pH-dependent miRNAs. Mean ± SEM, n=6-7.

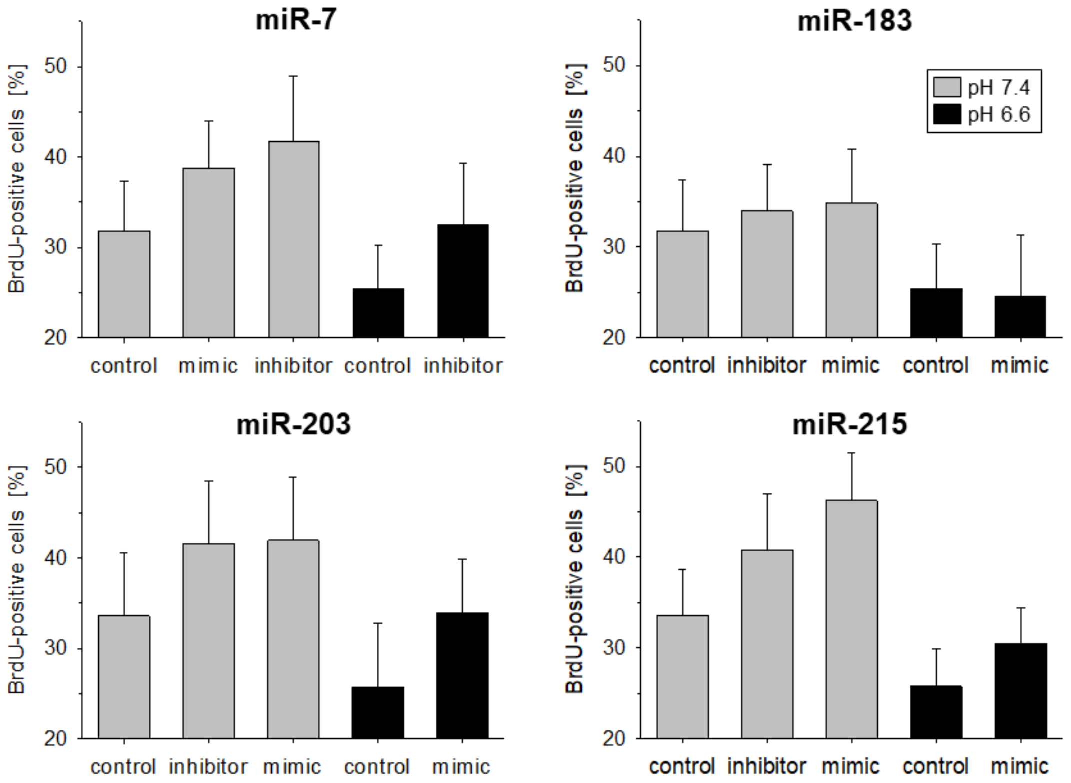

Fig. S5: Fraction of proliferating Walker-256 cells (BrdU uptake) after 24 h at pH 7.4 or 6.6 in combination with overexpression (mimic) or inhibition of pH-dependent miRNAs. Mean ± SEM, n=5-6.

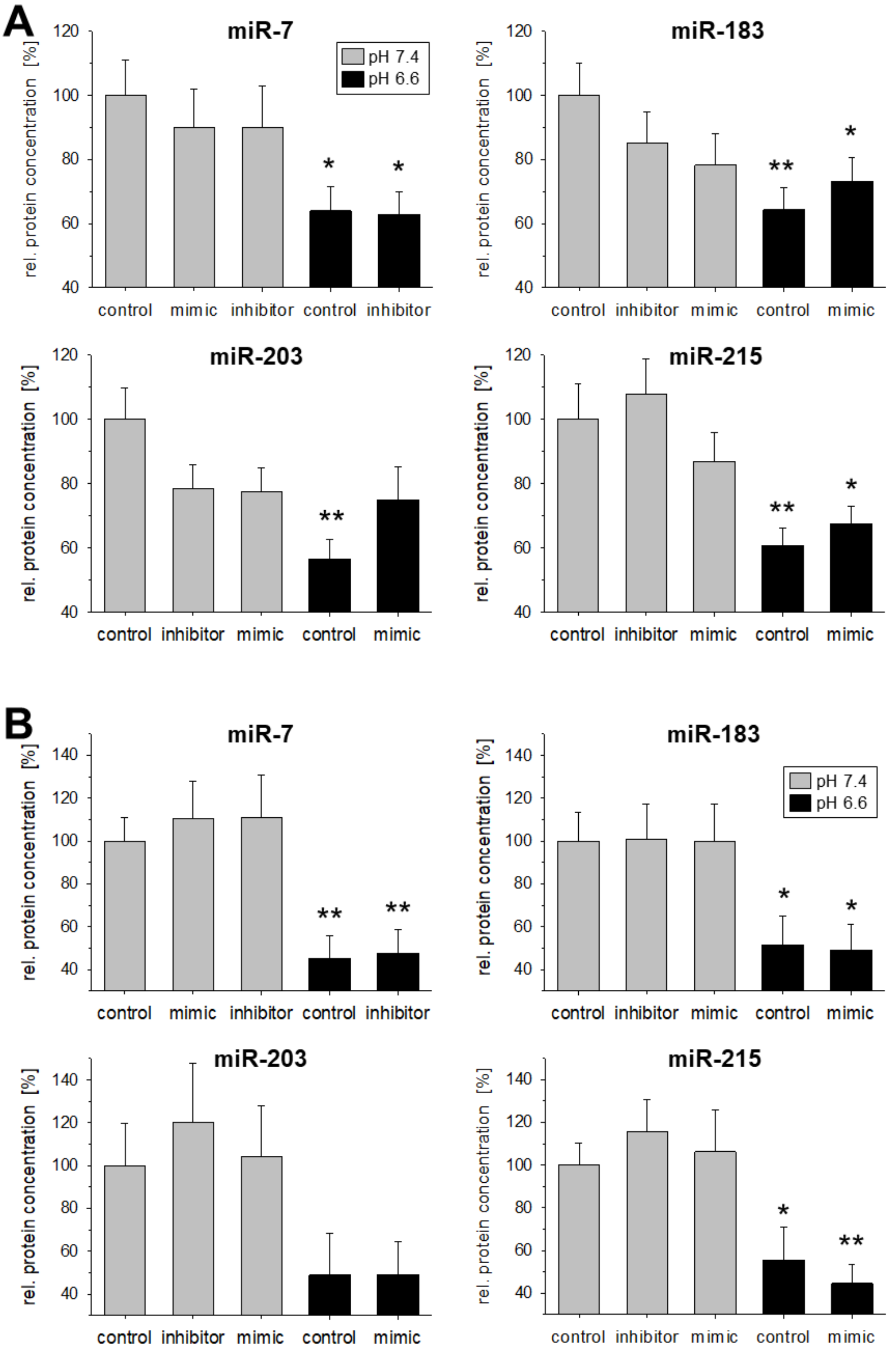

Fig. S6: Protein content (as a measure of cell number) of (**A**) AT1 cells or (**B**) Walker-256 cells after 24 h at pH 7.4 or 6.6 in combination with overexpression (mimic) or inhibition of pH-dependent miRNAs. Mean ± SEM, n=6-16, (*) p<0.05, (**) p<0.01 vs. pH 7.4 control.

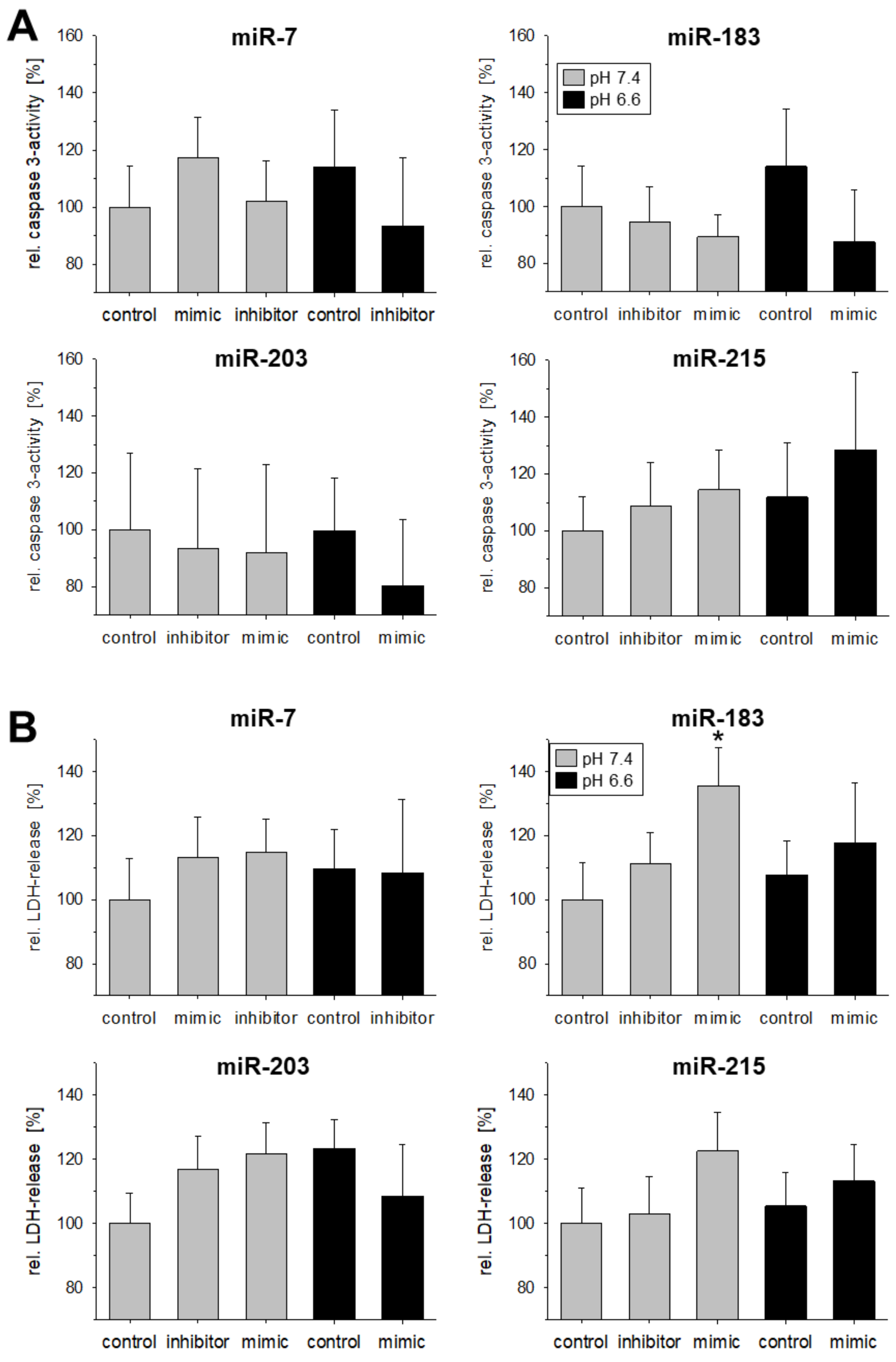

Fig. S7: (**A**) Apoptosis (caspase 3-activity) and (**B**) necrosis (LDH release) of AT1 cells after 24 h at pH 7.4 or 6.6 in combination with overexpression (mimic) or inhibition of pH-dependent miRNAs. Mean ± SEM, n=6-16, (*) $p<0.05$ vs. pH 7.4 control.

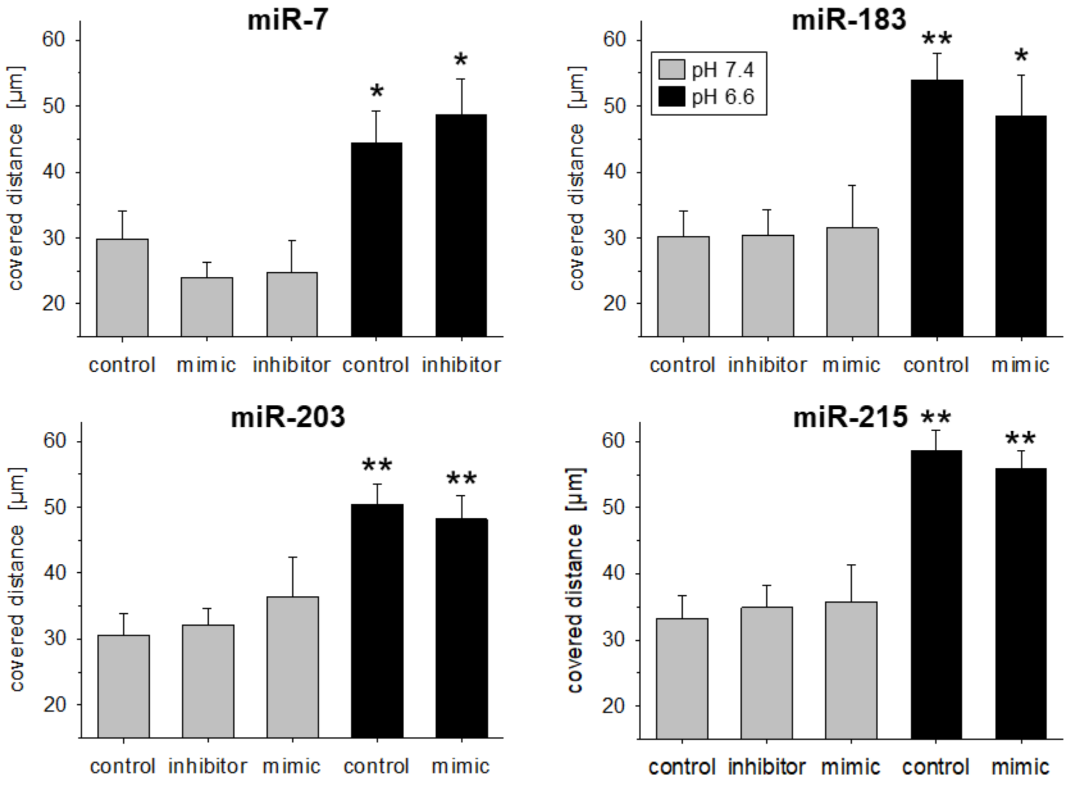

Fig. S8: Migration distance of AT1 cells after 24 h at pH 7.4 or 6.6 in combination with overexpression (mimic) or inhibition of pH-dependent miRNAs in AT1 cells. Mean ± SEM, n=5-14, (*) $p<0.05$, (**) $p<0.01$ vs. pH 7.4 control.
